# Left anterior cerebral artery arising from a right internal carotid artery: a rare case of carotid-anterior cerebral artery anastomosis

**DOI:** 10.1007/s00276-023-03218-4

**Published:** 2023-08-12

**Authors:** Talía Fuentes-Redondo, Luis-Alfonso Arráez-Aybar, Pedro Navia-Álvarez

**Affiliations:** 1 Department of Pediatrics, Complejo Hospitalario de Toledo, Toledo, Spain; 2 Department of Human Anatomy and Embryology, Faculty of Medicine, Complutense University of Madrid, 28070 Madrid, Spain; 3 Unit of Neuroradiology, La Paz University Hospital, Madrid, Spain

**Keywords:** Carotid – anterior cerebral artery anastomosis, Anterior cerebral artery, Internal carotid artery, Cerebral artery variation, Magnetic resonance angiography

## Abstract

Carotid-anterior cerebral artery anastomosis (carotid-ACA anastomosis) is described as infrequent vascular connections between the pre-ophthalmic segment of the internal carotid artery (ICA) and the A1 segment of the anterior cerebral artery (ACA). The embryological origin of these variant is still unclear and they are often associated to other vascular anomalies of the circle of Willis, as well as to the presence of aneurysms. Carotid-ACA anastomosis is often right-sided although left and bilateral cases have also been described. We report a rare case by MR angiography of a carotid-ACA anastomosis in which the abnormal vessel arises from the right ICA and takes an infraoptic course to join the A2 segment of the contralateral ACA, making this vascular anomaly function as a ‘left ACA with an origin at the right ICA’. The A1 segment of the left ACA is absent and both A2 segments of the ACAs present fenestration. To our knowledge, no similar cases have been reported in English literature so far.

## Introduction

Carotid-anterior cerebral artery anastomosis (carotid-ACA anastomosis) is extremely infrequent anatomical variations. In these cases, an anomalous vessel arises from the pre-ophthalmic segment of the internal carotid artery (ICA) to anastomose with the A1 segment of the anterior cerebral artery (ACA).

The first case of carotid-ACA anastomosis was described by Robinson in 1959 in an anatomical dissection [1]. After that, most cases were discovered by autopsy, surgery or angiography until the emergence of magnetic resonance angiography (MRA). Since then, cases reported by MRA are the most common due to its extended use and less invasiveness.

Real prevalence of this anatomical variation is unknown. Uchino et al. [2] reported an incidence in MR angiography of a 0.086%. No sex predominance has been recorded but right-sided carotid-ACA anastomosis predominance is well established. The reasons for this fact are still unclear. Various cases of left and bilateral variations have been reported but overall, these are quite rare [3–5]. Anomalies of the origin of the ophthalmic arteries are a frequent vascular variation associated with carotid-ACA anastomosis, among others [6].

## Case report: anatomic analysis

A 43-year-old female presented with recurrent episodes of headache with no clinical nor neurological findings at physical examination.

The anatomy of the case described was obtained by MR angiography with a non-contrast 3D TOF protocol, in a 1.5-Tesla scanner (Philips Medical Systems, Best, The Netherlands). Radiological images were enhanced with multiplanar reconstruction (MR), maximum intensity projection (MIP) and three-dimensional volume rendering (VR). Picture archiving and communication system (PACS) was used to evaluate images. One of the authors, an experienced neuroradiologist, reviewed all the images paying special attention to the anterior segment of the circle of Willis.

We present a case of a right carotid-ACA anastomosis in which the abnormal vessel arises from the right ICA, takes an infraoptic course, and eventually joins the A2 segment of the left ACA. The A1 segment of the left ACA is absent. In addition, both A2 segments of the ACAs present fenestration. No aneurysms are observed. The left ICA and middle cerebral artery (MCA) show no anomalies.

## Discussion

### Embryological aspects

The embryological origin of the carotid-ACA anastomosis is still a controversy. There are several theories that could explain the presence of this anomaly in adult vascular circulation, all of them are based on the persistence of an embryonic vessel. First example involves the enlargement and anastomosis of the prechiasmal branch of the ophthalmic artery, the superior hypophyseal branch of the ICA and the chiasmal branches of the ACA (‘prechiasmal anastomosis theory’). Second involves the persistence of the embryonic anastomosis between the primitive maxillary artery (which emerges from the cavernous ICA) and the anterior cerebral artery. Third is the persistence and enlargement of an embryological anastomotic loop between the primitive dorsal and ventral ophthalmic arteries (OAs) around the optic nerve [2, 5]. Around 4–8 mm embryonic stage, the ventral OA emerges from the ACA while the dorsal OA arises from the ICA. Both vessels play the role of the vascularization of the orbital region at this stage and merge together near the optic canal and the optic nerve. However, the adult OA is formed from caudal migration from the dorsal OA to the ventral OA. Then ventral OA regresses. The lack of regression of the ventral OA may explain the origin of carotid-ACA anastomosis [7]. The latter theory seems more probable as almost all of the carotid-ACA anastomosis emerge near the base of the OA and often associate anomalies of the origin of these ophthalmic arteries [5, 6].

### Anatomical aspects

Normally, ACA arises from the ICA and runs above the optic chiasm to anastomose with the contralateral ACA through the anterior communicating artery (ACoA). When a carotid-ACA anastomosis is present, an anomalous artery arises from the ICA at or close to the level of the OA, taking an infraoptic and prechiasmatic course to anastomose with the A1-A2 segment of the ACA, when they are present. This variation initially received the name of ‘infraoptic course of the ACA’ coined by Robinson in 1959 because the anomalous vessel misplaced the role of the ACA in this first case described. Nevertheless, as more cases were described, examples of a carotid-ACA anastomosis coexisting with a supraoptic A1 segment of the ACA were reported so the designation ‘carotid-ACA anastomosis’ seemed more accurate. In fact, both terms describe the anatomy of the vascular anomaly and are the most frequently found in English literature. Other keywords include ‘carotid siphon-pericallosal arterial anastomosis’, ‘interoptic course of the ACA’, and ‘preoptic origin of the ACA’ [8].

Wong et al. [9] made the first classification of carotid-ACA anastomosis considering the state of the A1 segment of the ACA into four types: type 1 has a normal A1 segment of the ACA, type 2 has an abnormal or absent ipsilateral A1 segment of the ACA, type 3 has abnormal or absent A1 segments bilaterally, type 4 has an accessory ACA. Type 2 is regarded as worse than type 1 and type 3 is the most severe type of variation [4, 6]. Our case cannot be accurately classified into any of these types of Wong et al. as the ipsilateral ACA has no anomalies and it is the A1 segment of the contralateral ACA which is absent. The carotid-ACA anastomosis arises from the right ICA but anastomoses with the A2 segment of the left ACA which can also be taken as if the left ACA arouse from the right ICA.

Carotid-ACA anastomosis is frequently related with other arterial variations near the circle of Willis. These might have correlation with an embryological failure of the normal development of this area during gestational period. Some anomalies described include ACA and ACoA fenestration, triplicated ACA, duplicated middle cerebral artery, hypoplasia of the contralateral vertebral artery, agenesia of contralateral ICA, fused pericallosal arteries [2, 3, 5, 9]. The current case showed fenestration of both A2 segments of the ACAs.

Special mention to the origin of the ophthalmic arteries (OAs) as it might vary when a carotid-ACA anastomosis is present. Either from the cavernous segment of the ICA to the sphenoidal branch of the middle meningeal artery, the OAs’ anomalous origin can be related with the embryological origin of both variants [4–6, 10]. In this case, there were no abnormalities in the origin of the OAs.

An increased incidence of aneurysms has been related to carotid-ACA anastomosis. Hemodynamic stress due to the anomalous blood flow can be the reason for this association. Its incidence is near 45% of the cases reported and they are usually found at the anterior part of the circle of Willis, mostly at the AcoA territory. Other territories affected by these aneurysms are ICA bifurcation, middle cerebral artery bifurcation, and basilar artery [2, 5, 7–9]. However, no aneurysms are observed in the present case.

### Clinical aspects

**Fig. 1:**
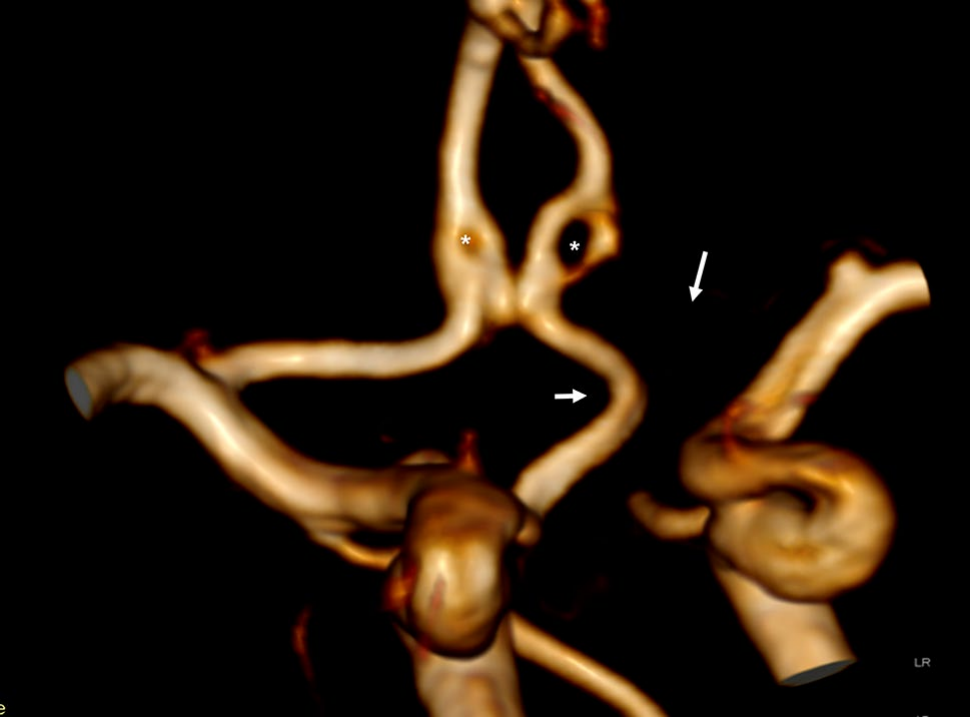
Magnetic resonance (MR) angiography with volume rendering (VR) image. Antero-posterior projection shows an abnormal vessel arising from the right internal carotid artery (ICA) to anastomose with the A2 segment of the left anterior cerebral artery (ACA) (short arrow). The A1 segment of the left ACA is absent (long arrow). A2 segments of both ACAs show fenestration (asterisks)

**Fig. 2:**
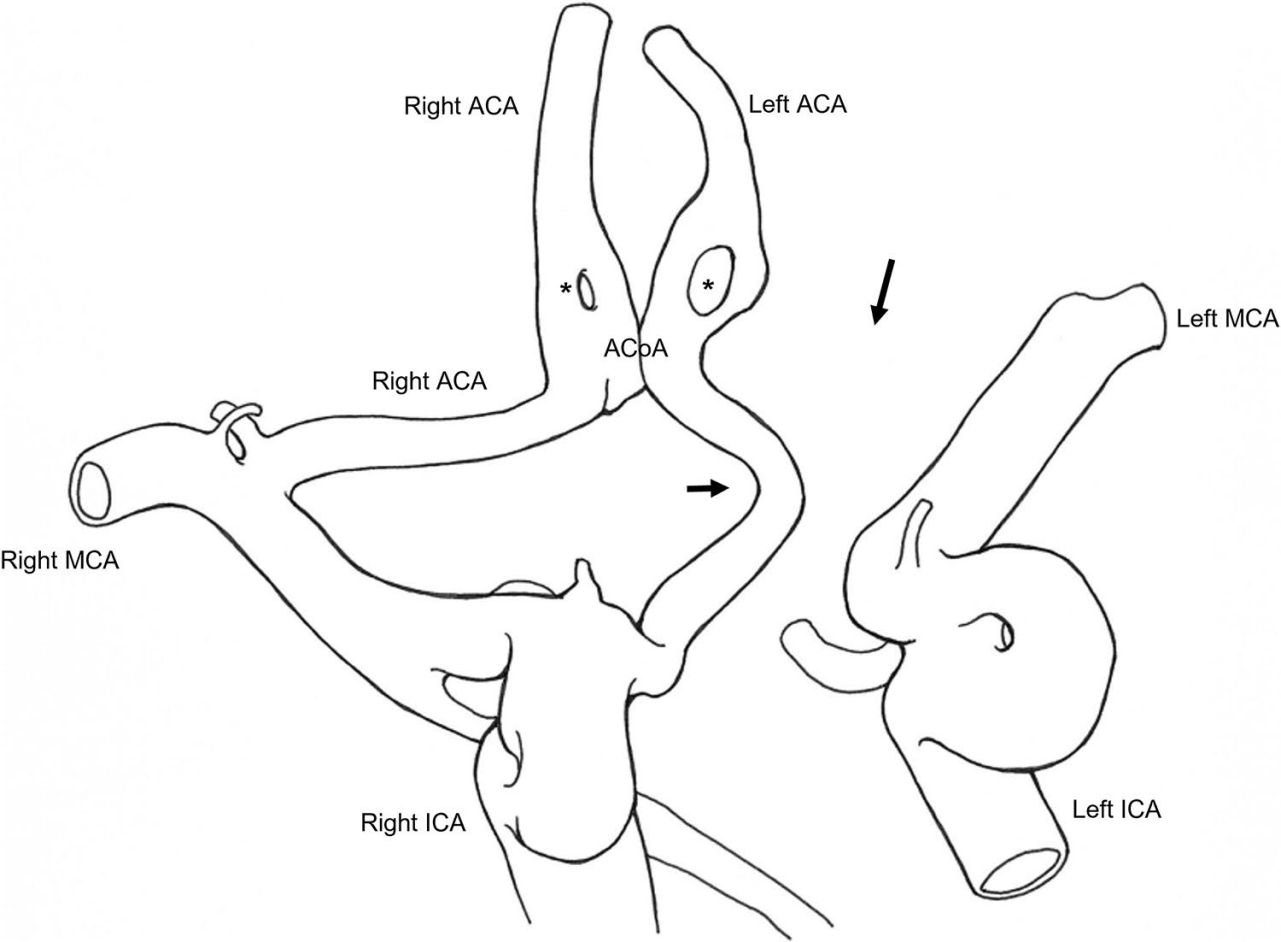
Schematic illustration of the present case. Fenestration of the ACA (asterisks). Carotid-ACA anastomosis (short arrow). Absent segment of the left ACA (long arrow). ACA anterior cerebral artery MCA middle cerebral artery ACoA anterior communicating artery ICA internal carotid artery

Clinical manifestations of the patients reported in literature so far are heterogeneous and may be not in relation with the presence of the carotid-ACA anastomosis itself. Cases described in MR angiography underwent imaging because of different reasons and carotid-ACA anastomosis were found incidentally. Headaches, mental and growth retardation, hemiparesia/hemiplegia are the main indications of cerebral imaging in the first place [8]. Few cases presented with visual symptoms because of compression of optic nerve or chiasm [7]. The imaging indication for this case was recurrent episodes of headache despite a normal physical and neurological examination (Figs. 1, 2).

Clinical importance of carotid-ACA anastomosis relies on the enhanced risk of aneurysms. Subarachnoid hemorrhage is frequently found in relation to aneurysms rupture. To prevent ischemic or hemorrhagic complications, a proper evaluation in advance of this kind of vascular anomalies is extremely important in those patients when undergoing clipping or coiling of AComA aneurysms. Besides, it is also important to evaluate the vascular anatomy before suprasellar surgery via the anterior cranial fossa due to the enhanced risk of injury of optic nerves and optic chiasm by proximity [2, 5–7] (Table 1).

**Table 1:** Carotid-ACA anastomosis cases referenced in this manuscript

Author (year)	No. of cases described	Side	Diagnostic modality	Anatomical findings
Robinson [1]	1	R	Autopsy	Absent right OA
Uchino et al. [8]	2	R R	MRA	Plexiform AcoA Contralateral A1 hypoplasia
Wong et al. [9]	2	B R	MRI	ICA aneurysm Parietal AVM
Uchino et al. [2]	3	R R R	MRA	ACA fenestration Triplicated ACA Duplicated MCA
Okano et al. [3]	1	L	MRA	Hipoplastic right VA
Yi et al. [5]	1	R	DSA	Paraclinoid aneurysm Ipsilateral OA originating from MMA
Uchino et al. [4]	1	B	MRA	OAs originating from MCAs
Uchino et al. [6]	1	B	MRA	OAs originating from the anastomotic arteries
Matsuura et al. [10]	1	R	MRA	Azygos ACA Ipsilateral OA originating from MMA
Kochar et al. [7]	2	B R	CTA MRA	Azygos ACA AcoA aneurysms
Present case	1	R	MRA	Anastomosis joins contralateral ACA Absent A1 segment of left ACA

*R* right *L* left *B* bilateral *MRA* magnetic resonance angiography *MRI* magnetic resonance imaging *DSA* digital subtraction angiography *CTA* computerized tomography angiography *OA* ophthalmic artery *AcoA* anterior communicating artery *ICA* internal carotid artery *AVM* arteriovenous malformation *ACA* anterior cerebral artery *MCA* middle cerebral artery *MMA* middle meningeal artery *VA* vertebral artery

## Conclusion

We reported a rare case by MR angiography of a right carotid-ACA anastomosis consisting in an abnormal vessel arising from the right ICA to join the left A2 segment of the contralateral ACA, operating as a left ACA itself. The regular origin of the A1 segment of the left ACA was absent. No other anatomical variations were described except for fenestration of both A2 segments of the ACAs. No aneurysms were found. We believe this is the first case described of carotid-ACA anastomosis with these features in English literature.

## Data Availability

Not applicable.
